# Immune checkpoint inhibitors for patients with microsatellite instability-high colorectal cancer: protocol of a pooled analysis of clinical trials

**DOI:** 10.3389/fonc.2023.1331937

**Published:** 2024-01-03

**Authors:** Xiaojun Tang, Xinshu Xu, Ruobing Chen, Mengmeng Zhang, Zubing Mei, Shuangxi Zhang

**Affiliations:** ^1^ Department of Spinal Surgery, The Second Affiliated Hospital, Hengyang Medical School, University of South China, Hengyang, Hunan, China; ^2^ Department of Anorectal Surgery, The First Affiliated Hospital of Henan University of Chinese Medicine, Zhengzhou, Henan, China; ^3^ First Clinical Medical College, Henan University of Chinese Medicine, Zhengzhou, Henan, China; ^4^ Department of Anorectal Surgery, Shuguang Hospital, Shanghai University of Traditional Chinese Medicine, Shanghai, China; ^5^ Anorectal Disease Institute of Shuguang Hospital, Shanghai, China

**Keywords:** immune checkpoint inhibitors, microsatellite instability-high, colorectal cancer, clinical trials, pooled analysis

## Abstract

**Introduction:**

Colorectal cancer (CRC) is the third most common cause of cancer and the second leading cause of cancer-related deaths worldwide. Microsatellite instability-high (MSI-H) is a distinct molecular subtype of CRC that occurs in approximately 15% of all cases. Recently, immune checkpoint inhibitors (ICIs) have emerged as a promising therapeutic approach for patients with MSI-H colorectal cancer, exhibiting higher response rates than standard chemotherapies. To assess the effectiveness and safety of ICIs for the treatment of patients with MSI-H CRC, we propose a comprehensive pooled analysis of clinical trial data.

**Methods and analysis:**

A systematic search of multiple electronic databases, including PubMed, EMBASE, Cochrane Library, and Clinicaltrials.gov, will be conducted from their inception until September, 2023 to identify eligible randomized controlled trials (RCTs) and non-randomized studies. Inclusion criteria comprise studies of adult patients with histologically confirmed MSI-H CRC treated with immune checkpoint inhibitors, with a comparison to a control group receiving conventional therapies. Outcomes of interest will be overall survival (OS), progression-free survival (PFS), objective response rate (ORR), disease control rate (DCR), and incidence of treatment-related adverse events (AEs). The Cochrane Risk of Bias tool and the Risk of Bias in Non-randomized Studies of Interventions (ROBINS-I) tool will be employed to evaluate the methodological quality of included studies. A random-effects model using the DerSimonian and Laird method will be applied for pooling the effect estimates, calculating hazard ratios (HRs) or risk ratios (RRs) with their corresponding 95% confidence intervals (CIs). Heterogeneity will be assessed using I² statistics, and subgroup analysis and meta-regression will be performed to explore potential effect modifiers in case of substantial heterogeneity. Publication bias will be evaluated with funnel plots and Egger’s test. Sensitivity analysis will be conducted to assess the robustness of the results.

**Discussion:**

This meta-analysis will synthesize available evidence from clinical trials on immune checkpoint inhibitors in treating MSI-H colorectal cancer. The findings will offer valuable information about the effectiveness and safety of ICIs in this patient population, contributing to the refinement of clinical guidelines and enhancing the decision-making process for healthcare providers, policy-makers, and patients. The comprehensive analysis of subgroups and sensitivity allows for an in-depth understanding of potential effect modification, providing essential directions for future research.

**Ethics and dissemination:**

This study will involve the use of published data; hence, ethical approval is not required. The results of the study will be disseminated through publications in peer-reviewed journals and presentations at relevant conferences. The findings will potentially impact clinical decision-making and contribute to the development of evidence-based treatment recommendations for patients with MSI-H colorectal cancer.

**Clinical trial registration:**

Open Science Framework identifier, 10.17605/OSF.IO/ZHJ85

## Introduction

Colorectal cancer (CRC) remains a leading cause of cancer-related death worldwide (1). Over the past few decades, CRC has become the third most commonly diagnosed cancer globally, with an estimated 1.9 million new cases and 935,000 deaths reported in 2020 (2, 3). With the advent of novel targeted and immunotherapeutic agents, the therapeutic landscape for patients with CRC has changed dramatically, transforming the management and outcomes of the disease (4–6). Among the biomarkers of particular interest in guiding the direction of CRC treatment are microsatellites, which are short tandem repeats of DNA found throughout the genome. Microsatellite instability (MSI) is a known predictor of CRC patient response to treatment, with high levels of MSI (MSI-H) being identified in approximately 15% of CRC cases and being associated with distinct clinicopathological features (7, 8).

Recently, the emergence of immune checkpoint inhibitors (ICIs) has revolutionized cancer care, resulting in favorable outcomes for various malignancies. ICIs target inhibitory pathways in T-cells, such as the programmed death-1 (PD-1)/programmed death-ligand 1 (PD-L1) and cytotoxic T-lymphocyte-associated antigen 4 (CTLA-4) axes, thereby augmenting the anti-tumor immune response. In patients with MSI-H CRC, known to have a high tumor mutation burden and immunogenic antigens, ICIs have demonstrated differential response compared to their MMR-proficient (MMR-P) counterparts (9). Several clinical trials have investigated the efficacy and safety of ICIs in patients with MSI-H CRC, including well-designed phase II and III trials assessing the activity of PD-1 inhibitors and serving as a late-line treatment option in the management of MSI-H CRC, such as nivolumab and pembrolizumab, both as monotherapy and in combination with other agents (10–13). As a result, these checkpoint inhibitors have received regulatory approval for the management of patients with MSI-H CRC, especially in those who have experienced treatment failure with conventional chemotherapeutic regimens.

Despite the clinical promise of ICIs in patients with MSI-H CRC, approximately 30% of patients still experience early disease progression, indicating treatment resistance (14). However, the lack of large-scale, well-conducted, and homogeneous systematic reviews and meta-analyses has limited the quantitative assessment of the benefits and harms associated with ICI utilization in this patient population. This knowledge gap highlights the need for a comprehensive and evidence-based evaluation to support informed decision-making in the clinical management of patients with MSI-H CRC.

Therefore, the present study protocol aims to outline the methodology and analysis plan for a pooled analysis of clinical trials investigating the outcomes of ICI treatment for patients with MSI-H CRC, emphasizing the efficacy, safety, and response rate in the context of both monotherapy and combination therapy regimens. When available, we will also analyze individual patient data from these trials to derive meaningful conclusions. The objective of this protocol article is to provide transparency, prevent outcome reporting bias, and document the study plan before conducting the analysis. Through this comprehensive and evidence-based evaluation, we seek to deliver a relevant and reliable assessment of the therapeutic potential and drawbacks of ICIs in MSI-H CRC, thereby informing clinical practice and guiding future research endeavors in this field.

## Methods

### Study registration

To ensure transparency, prevent outcome reporting bias, and document the study protocol, this pooled analysis will be conducted following the Preferred Reporting Items for Systematic Reviews and Meta-Analyses Protocol (PRISMA-P) guidelines (15) (**Supplementary Table 1**). The study has been prospectively registered at the Open Science Framework (OSF) website with the registration number of DOI 10.17605/OSF.IO/ZHJ85. The registration was completed before the commencement of data collection and analysis. The registered full study protocol, which outlines the research question, inclusion and exclusion criteria, data extraction procedures, and statistical analysis plan, can be accessed on the OSF website at https://osf.io/xugps.

### Data sources and search strategies

To identify relevant studies investigating the efficacy of immune checkpoint inhibitors for patients with MSI-H colorectal cancer, we will develop a comprehensive search strategy. This search strategy will be conducted across electronic databases, including PubMed, Embase, Cochrane Library, and ClinicalTrials.gov, from inception to September 2023. We will combine Medical Subject Headings (MeSH) terms and free-text words related to immune checkpoint inhibitors, MSI-H colorectal cancer, and clinical trials. The search terms will be (“immune checkpoint inhibitors” OR “PD-1 inhibitors” OR “PD-L1 inhibitors” OR “CTLA-4 inhibitors”) AND (“microsatellite instability-high” OR “MSI-H” OR “colorectal cancer”). We will apply the filters of “Clinical Trial” and “Humans” to ensure the relevance of the studies to our research question. The detailed search strategy of a major database is presented in **Table 1**.

**Table 1:** Search Strategy for Pubmed Database.

Colorectal Cancer terms:
1 “Colorectal Neoplasms”[Mesh]
2 “Neoplasms”[Mesh]
3 (carcinoma* OR neoplas* OR tumour* OR tumor* OR cancer* OR oncolog* OR malignan* OR carcinogen* OR oncogen*)[Title/Abstract]
4 2 OR 3
5 (colorectal* OR colon* OR rectal*)[Title/Abstract]
6 4 AND 5
7 1 OR 6
Immune Checkpoint Inhibitor terms:
8 “Immune Checkpoint Inhibitors”[Mesh]
9 “Cell Cycle Checkpoints”[Mesh]
10 “CTLA-4 Antigen”[Mesh]
11 (check-point inhibitor* OR checkpoint inhibitor* OR CTLA-1 OR CTLA-4 OR cytotoxic T-lymphocyte-associated protein 4 OR PD-L1 OR PD-1 OR programmed death receptor 1 OR immune checkpoint inhibitor OR ipilimumab OR tremelimumab OR nivolumab OR pembrolizumab OR durvalumab OR atezolizumab OR cemiplimab OR spartalizumab OR MED10680 OR AMP-224 OR pidilizumab OR atezolimab OR MED14736 OR avelumab OR BMS-936559 AND durvalumab OR MEDI4736) [Title/Abstract]
12 OR/8-11
Microsatellite Instability terms:
13 “Microsatellite Instability”[Mesh]
14 “DNA Mismatch Repair”[Mesh]
15 (microsatellite instability or MSI-H or MSI or mismatch-repair deficient or microsatellite repeats or mismatch repair or replication error or BAT25 or BAT26 or D5S346 or D2S123 or D17S250 or dMMR or MLH1 or MSH2 or MSH6 or PMS2) [Title/Abstract]
16 OR/13-15
Final search results: Combined:
17 7 AND 12 AND 16

### Selection criteria

To ensure the quality and relevance of studies included in this pooled analysis, we will apply the following “PICOS” framework criteria:

- Study design: Only clinical trials, including randomized controlled trials (RCTs) and non-randomized controlled trials, phase 2 clinical trials will be considered for inclusion.
- Participants: Studies involving adult patients diagnosed with MSI-H colorectal cancer will be included.
- Intervention: Studies evaluating the use of immune checkpoint inhibitors (such as PD-1 inhibitors, PD-L1 inhibitors, or CTLA-4 inhibitors) as a treatment modality will be eligible for inclusion.
- Outcome measures: Studies reporting clinical outcomes, including but not limited to overall response rate, progression-free survival, and overall survival, will be considered for inclusion. Overall response rate (ORR) is defined as the proportion of colorectal cancer patients who experience either a complete response (disappearance of all lesions) or a partial response (a significant reduction in the size of the lesions) to treatment. Progression-free survival (PFS) is defined as the duration of time from the start of treatment (or enrollment in a clinical trial) until disease progression or death from any cause. While overall survival (OS) is defined as the time from the start of treatment or diagnosis to the date of death from any cause.

### Exclusion criteria

Studies will be excluded if they meet any of the following criteria:

1) Studies that do not evaluate the use of ICIs as a treatment modality for patients with MSI-H CRC or studies that do not report relevant outcomes such as ORR, PFS, or OS; 2) Studies conducted on animals or *in vitro*; 3) Case reports, review articles, editorials, meta-analyses, and conference abstracts.

### Study selection

To ensure the quality and relevance of studies included in this systematic review and meta-analysis, a systematic approach will be employed for the selection of studies eligible for inclusion. Two independent reviewers will screen the titles and abstracts of all identified studies based on predefined inclusion and exclusion criteria. Potentially eligible studies will have their full texts retrieved and reviewed for final inclusion.

### Data collection and extraction

A standardized data collection form will be developed to systematically extract relevant information from the included studies. Two independent reviewers will use the predefined form to extract data from each study. Any discrepancies or disagreements will be resolved through discussion or consultation with a third reviewer. The following information will be extracted from each included study:

- Study Characteristics: First authors, publication year, trial name, study design, duration of follow-up, and geographical location.
- Participant Characteristics: Patient demographics (age, gender), sample size, tumor stage, MSI-H status, and prior treatments.
- Intervention: Type of immune checkpoint inhibitors used, dosages, treatment regimens, and duration.
- Comparison for RCTs: Type of comparison agents and dosages.
- Outcomes: Clinical outcomes reported, including overall response rate, progression-free survival, and overall survival.

### Risk of bias assessment

To ensure the quality and rigor of the included studies, the methodological quality and risk of bias for each study will be assessed independently by two reviewers using appropriate tools based on the study design. Discrepancies will be resolved through discussion or consultation with a third reviewer.

For RCTs, we will employ the Cochrane Risk of Bias tool to assess the risk of bias (16). We will evaluate the following seven domains: random sequence generation, allocation concealment, blinding of participants and personnel, blinding of outcome assessment, incomplete outcome data, selective reporting, and other bias. We will rate each domain as “low risk,” “high risk,” or “unclear risk” of bias based on the information provided in the original study publication. For non-RCTs, we will utilize the Risk of Bias in Non-Randomized Studies of Interventions (ROBINS-I) tool (17). This tool assesses the risk of bias across seven domains, including the bias due to confounding, selection of participants, classification of interventions, deviation from intended interventions, missing data, measurement of outcomes, and selection of the reported result. We will rate each domain as “low risk,” “moderate risk,” “serious risk,” “critical risk,” or “no information” based on the assessment of bias within that domain.

### Grading of evidence

To evaluate the quality and strength of evidence for outcomes assessed in this study, we will use the Grading of Recommendations Assessment, Development, and Evaluation (GRADE) approach (18). The GRADE methodology considers factors such as study design, risk of bias, consistency of results, precision, and other considerations when assigning a level of evidence to each outcome. Two independent reviewers will assess the quality of evidence for each outcome across included studies based on GRADE criteria. The initial quality level of evidence will be determined as high, moderate, low, or very low. The level could be downgraded for factors such as risk of bias, inconsistency, imprecision, indirectness, and publication bias. Conversely, the level could be upgraded for a large magnitude of effect, dose-response gradient, and plausible confounding. Discrepancies in grading will be resolved through discussion or consultation with a third reviewer to reach a consensus. The final quality of evidence for each outcome will be summarized using GRADE tables. These tables present the quality of evidence along with a brief explanation of the reasons for downgrading or upgrading, if applicable.

### Statistical analysis

All statistical analyses will be conducted using Stata statistical software (version 15.0; Stata Corp, College Station, TX). For dichotomous outcomes, the pooled risk ratio (RR) and its corresponding 95% confidence interval (CI) will be calculated using the Mantel-Haenszel method (19). Continuous outcomes will be synthesized using weighted mean difference (WMD) or standardized mean difference (SMD) with 95% CI (20). Forest plots will be generated to visually represent the effect sizes and their uncertainties.

Due to the potential variations in study populations, ICI types, and outcome domain across trials, a random-effects model will be employed for the meta-analysis to account for potential heterogeneity. Heterogeneity among included trials will be assessed using the I^2^ statistic, which quantifies the proportion of total variation attributed to between-study heterogeneity. An I^2^ value >50% will be considered substantial heterogeneity (21, 22).

### Subgroup analyses

In cases of significant heterogeneity, subgroup analyses will be performed to explore potential sources of heterogeneity based on relevant study characteristics such as study design, type of immune checkpoint inhibitor, and patient demographics. We recognize the potential differences in responses to immune checkpoint inhibitors in patients at different cancer stages. Therefore, subgroup analyses will be performed to explore the treatment effects according to the stage of colorectal cancer (early-stage versus advanced-stage disease). We will attempt to stratify the studies based on these stages, provided sufficient data is available in the trials. Additionally, we will present forest plots for each subgroup to visually represent the effect size estimates and their uncertainty. Meta-regression will also be performed to examine the potential impact of covariates on the effect size.

Potential publication bias will be assessed visually using funnel plots and statistically using Egger’s regression test. The presence of publication bias will be suggested by asymmetry in the funnel plot or a significant p-value (<0.05) from Egger’s test (23). In case of the presence of publication bias, the trim and fill analysis will be used to assess and adjust this bias (24). This analysis estimates the number of missing studies that may not have been published due to small sample sizes or non-significant results by imputing these missing studies and recalculating the effect size. To evaluate the robustness of the meta-analysis results, sensitivity analyses will be conducted by excluding studies with high risk of bias or small sample sizes individually to assess their influence on the overall effect estimates.

### Ethics and dissemination

Given the nature of our study design, ethical approval is not required. We will disseminate the findings from our study by presenting them at relevant conferences and publishing the final report in a peer-reviewed journal.

## Discussion

Our study will provide compelling evidence regarding the efficacy of immune checkpoint inhibitors in treating patients with MSI-H colorectal cancer. The primary outcome measures, including overall response rate and progression-free survival, will exhibit the real and comprehensive effectiveness among patients receiving immune checkpoint inhibitor therapy.

The potential effectiveness of immune checkpoint inhibitors in MSI-H colorectal cancer patients can be attributed to the unique molecular characteristics of this subgroup. The hypermutated nature of MSI-H tumors results in a higher neoantigen load, rendering them more immunogenic. This phenomenon leads to increased recognition by immune cells, facilitating enhanced antitumor immune responses (7, 12, 25).

Our study will have several potential strengths. Firstly, our study is the first and most comprehensive pooled analysis to address the outcomes of ICIs for MSI-High colorectal cancer. Moreover, we will employ a meticulous search strategy across multiple reputable databases, ensuring a comprehensive inclusion of relevant studies (26, 27). Secondly, we will adhere to robust methodology by following established guidelines for study selection, data extraction, and statistical analyses, enhancing the reliability of our findings (28). Thirdly, rigorous assessment of risk of bias and grading of evidence using the GRADE approach will enhance the quality of our synthesized results (29). Fourthly, the inclusion of clinical trials involving actual patients will ensure the applicability of our conclusions to real-world scenarios (30). Finally, by pooling data from multiple trials, we will achieve increased statistical power, allowing for more precise effect estimates regarding the real effect estimates of ICI in the treatment of MSI-H colorectal cancer (31).

However, there will also be potential limitations to this study. Firstly, variability in study designs, patient characteristics, and treatment protocols may contribute to heterogeneity, which will influence our pooled effect estimates. Secondly, despite our efforts to minimize publication bias, it remains a potential limitation, as positive results are more likely to be published. Thirdly, most of the included trials provide only short-term follow-up data, limiting our ability to assess long-term survival outcomes and treatment durability. Fourthly, it is possible that the lack of detailed individual patient data from the included trials could restrict our ability to perform extensive subgroup analyses and explore potential modifiers of treatment effect. Finally, the inclusion of both randomized controlled trials and non-randomized studies may introduce bias due to differing study designs and potential confounding.

Our findings suggest that immune checkpoint inhibitors may be a promising treatment option for MSI-H colorectal cancer patients. The observed improvements in response rates and progression-free survival underscore the potential clinical impact of these therapies. Clinicians should incorporate immune checkpoint inhibitors into the treatment armamentarium for MSI-H colorectal cancer patients. However, patient selection and appropriate combination therapies warrant further investigation. Future studies should explore biomarkers predictive of response and resistance, allowing for personalized treatment strategies (11, 32).

## Data availability statement

The original contributions presented in the study are included in the article/**Supplementary Material**. Further inquiries can be directed to the corresponding author.

## Author contributions

XT: Conceptualization, Methodology, Validation, Writing – original draft. XX: Methodology, Validation, Writing – original draft. RC: Writing – original draft. MZ: Methodology, Validation, Writing – original draft. ZM: Conceptualization, Methodology, Supervision, Validation, Writing – original draft, Writing – review & editing. SZ: Conceptualization, Investigation, Methodology, Software, Validation, Writing – original draft.

## Supplementary material

Supplementary Table 1 PRISMA-P checklist.
